# A Deep Reconstruction Framework with Ringing Artifact Suppression for Overexposed Remote Sensing Image Restoration

**DOI:** 10.3390/jimaging12090403

**Published:** 2026-08-26

**Authors:** Dinghao Yang, Yujie Xing, Hongmei Li, Xuquan Wang, Xiong Dun

**Affiliations:** 1 Institute of Precision Optical Engineering, School of Physics Science and Engineering, Tongji University, Shanghai 200092, China; 2332691@tongji.edu.cn (D.Y.); 2511445@tongji.edu.cn (H.L.); dunx@tongji.edu.cn (X.D.); 2 MOE Key Laboratory of Advanced Micro-Structured Materials, Shanghai 200092, China; 3 Shanghai Frontiers Science Center of Digital Optics, Shanghai 200092, China

**Keywords:** computational imaging, ringing artifacts, overexposed scenes, image restoration, deep learning-based reconstruction

## Abstract

Computational imaging shifts part of the aberration correction from optical hardware to algorithms, offering a viable path toward compact, simplified systems. However, overexposed regions—often caused by phenomena such as water-body reflections—can readily induce severe ringing artifacts in reconstructed images. To address this problem, we propose a Ringing-perceptive Cooperative Reconstruction Network (RPCR-Net). This network integrates a learned Wiener filter and a field-of-view shared kernel prediction network (FOV-KPN) for feature extraction and innovatively incorporates a combined regularization mechanism that leverages a Local Maximum Gradient Prior and a multi-scale ringing measurement model within its loss function to suppress artifacts while preserving details. Validated on a constructed overexposed image dataset, RPCR-Net improves the Peak Signal-to-Noise Ratio (PSNR) from 29.08 dB to 37.06 dB and the Structural Similarity Index Measure (SSIM) from 0.8795 to 0.9549. Experiments on real-world scenes further confirm its capability to suppress ringing artifacts while maintaining visual quality. The proposed method can generate high-quality images such as image reconstruction and robustness improvement in optical systems.

## 1. Introduction

High-quality optical imaging is essential for a wide range of remote sensing interpretation tasks, such as land-cover pixel classification and content-based image retrieval [1,2,3]. Conventional remote sensing optical systems generally rely on multi-element lens assemblies [4] and high-precision manufacturing processes [5] to correct optical aberrations and ensure high imaging quality. However, these designs inevitably lead to bulky and massive payloads, imposing significant limitations on satellite and unmanned aerial vehicle platforms that increasingly demand miniaturized and lightweight configurations. In recent years, computational imaging has emerged as a transformative paradigm that co-designs optics and reconstruction algorithms, thereby shifting a portion of aberration correction from the optical hardware to the computational domain. This facilitates the development of compact remote sensing cameras with imaging performance comparable to that of traditional, bulkier optical systems, and has attracted considerable attention within the remote sensing community [6,7]. Despite these advantages, computational imaging systems remain susceptible to extreme illumination conditions frequently encountered in Earth observation. In particular, when imaging scenes containing specular highlights, sun glint over water bodies, or highly reflective urban structures, overexposed regions can induce severe ringing artifacts in the reconstructed images, substantially degrading their radiometric and geometric quality [8,9,10]. Consequently, endowing compact computational imaging systems with robustness and adaptability against overexposed remote sensing scenes—while preserving high-fidelity imagery for downstream analysis—remains an outstanding challenge.

Ringing artifacts arise from nonlinear response and signal truncation caused by exceeding the sensor’s dynamic range, which violates the energy conservation assumption underlying linear imaging models [11]. Existing approaches for mitigating ringing artifacts can be broadly divided into two categories. The first category detects and processes overexposed regions independently—for example, by applying tailored weights or models to saturated pixels via classification or regional decoupling—to reduce their influence on reconstruction [12,13,14,15]. However, these methods cannot compensate for energy distortion that has already propagated into neighboring regions during the blurring process, limiting their ability to fundamentally eliminate ringing artifacts. The second category targets the ringing patterns themselves, employing strategies such as the Local Maximum Gradient (LMG) prior [16] or a ringing measurement model [17] to constrain image gradients. Nevertheless, these approaches often face a trade-off: while they suppress ringing, they may over-smooth genuine high-gradient structures or fail to adequately address subtle ringing effects. Consequently, achieving a satisfactory balance between artifact suppression and detail preservation remains challenging.

In recent years, deep neural networks have become increasingly dominant in image reconstruction due to their strong capacity for modeling nonlinear degradations and have been extensively applied to spatially varying optical aberrations. The evolution from Res-Unet, designed for mild point spread function (PSF) variations [18], to a field-of-view shared kernel prediction network (FOV-KPN), which leverages dynamic convolutions to handle more severe spatial variability [19], and further to the recently proposed learned Wiener-based FOV-KPN neural network [20], and more recently, transformer-based architectures such as Uformer [21], Restormer [22], and the simple yet effective baseline NAFNet [23] have achieved state-of-the-art performance on various image restoration tasks. Despite these advances, a critical limitation remains: current networks often fail to effectively suppress the ringing artifacts prevalent in overexposed scenes. This is primarily because saturated pixels introduce severe local nonlinearities that violate the assumptions of conventional linear degradation models. Moreover, existing network architectures and loss functions are not explicitly designed to model or correct such artifacts, resulting in degraded reconstruction performance. These shortcomings underscore the insufficient robustness of contemporary deep learning-based computational imaging methods under challenging imaging conditions.

To address this issue, we propose a Ringing-Perceptive Cooperative Reconstruction Network (RPCR-Net). RPCR-Net adopts the learned Wiener-based FOV-KPN architecture [20] as its backbone, but its key novelty lies in a synergistic ringing-perceptive loss function. By exploiting the complementary limitations of the LMG prior [16] (strong ringing suppression but tendency to over-smooth details) and the ringing measurement model [17] (good detail preservation but weak sensitivity to mild ringing), we design a loss that combines both priors in a cooperative manner: the LMG term enforces robust ringing removal, while the ringing measurement term protects genuine image structures from being mistakenly penalized. This co-design enables RPCR-Net to effectively suppress ringing artifacts while preserving fine details, a capability that neither prior alone nor the original reconstruction network could provide. To validate the proposed method, we constructed a simulated dataset of overexposed images and conducted reconstruction experiments. On this dataset, RPCR-Net improves the PSNR from 34.76 dB (achieved by the baseline learned Wiener-based FOV-KPN) to 37.06 dB and increases the SSIM from 0.9459 to 0.9549. Furthermore, validation on an actual prototype demonstrates that the method achieves superior ringing suppression and detail preservation visually. These results highlight the improved robustness of computational imaging methods in real-world scenarios, paving the way for their practical deployment in remote sensing applications.

## 2. Related Works

### 2.1. Ringing Artifact Suppression Methods

Ringing artifacts are a common type of image artifact in computational imaging, originating from the nonlinear response of an optical system. Specifically, the detector has an upper saturation limit. When ambient light intensity is excessively high, the irradiance on certain pixels may far exceed this limit, resulting in signal truncation and a nonlinear response. In subsequent image reconstruction algorithms, such nonlinear responses interfere with the reconstruction process, ultimately manifesting in the image as abnormal oscillatory stripes along edges or high-contrast regions, which are known as ringing [11].

Studies by Fergus [12], Cho [13], and Tai [14] have shown that when environmental light intensity exceeds the upper limit of a sensor’s dynamic range, signal truncation occurs in certain areas due to response saturation, leading to a nonlinear response in the imaging system. Such nonlinear effects disrupt the energy conservation relationship between overexposed regions and their neighboring pixels. In the subsequent deconvolution process, this energy mismatch acts as a source of high-frequency oscillation, which typically manifests as ringing artifacts in the reconstructed image.

A common strategy to mitigate ringing reduces the influence of overexposed regions during reconstruction, since nonlinear distortion originates from saturated pixels. Accordingly, existing work introduces specially designed regularization terms in model-based iterative reconstruction algorithms. Harmeling et al. proposed a ringing suppression method via saturated pixel detection [15], assigning lower weights to saturated regions and higher weights to normal ones, thereby excluding overexposed areas from the objective function and achieving optimal weighting through a dynamic mechanism. Under traditional Total Variation regularization, this method raises the reconstruction PSNR from 18.56 dB to 21.91 dB. Whyte et al. proposed a ringing suppression method based on image region decoupling [8]. At each iteration, the image is divided into overexposed and non-overexposed regions; for overexposed regions, a continuously differentiable saturation response function approximates the forward model, reducing reliance on saturated pixels and avoiding ringing caused by forcibly fitting nonlinear truncation, while non-overexposed regions undergo standard restoration. Additionally, a regularization term smooths the transition zone between the two regions, effectively suppressing residual ringing. These methods aim to suppress ringing by isolating or down-weighting overexposed regions, implicitly assuming this suffices to prevent the propagation of nonlinear distortions.

Although the above methods suppress ringing by identifying and separately processing overexposed regions, they share a fundamental limitation: the actual energy corresponding to overexposed areas has already diffused into surrounding regions during the blurring process. These methods cannot correct the resulting energy distribution distortion in neighboring areas, making it difficult to fundamentally eliminate ringing. In other words, by the time the reconstruction stage begins, the adverse effects of saturation have already spread beyond the overexposed regions themselves, rendering region-specific processing inherently insufficient.

In light of this limitation, an alternative line of research has shifted focus from identifying overexposed regions to directly identifying and suppressing the ringing artifacts themselves. This approach treats ringing as a pattern to be explicitly constrained, independent of its root cause. Chen et al. proposed a non-blind restoration method based on the LMG prior [16]. By establishing a quantitative relationship between local image gradient extremes and ringing intensity, this method incorporates gradient information into a regularization constraint, achieving ringing suppression through iterative optimization. Experiments showed that this method improved PSNR from 29.08 dB to 30.37 dB. However, because it essentially penalizes high local gradient values, it tends to over-smooth genuine structural gradients—such as rich textures or sharp edges—compromising the reconstruction of high-frequency details. A different strategy was proposed by Ali Mosleh et al., who developed a non-blind restoration method based on a ringing measurement model [17]. This method suppresses ringing by minimizing the absolute deviation between the gradients of the reconstructed image and the blurred image (BL). Experiments showed that compared to previous methods, it improved PSNR from 22.39 dB to 24.30 dB. Nevertheless, while this method preserves high-frequency details, its use of gradient operators introduces a gradient amplification effect. As a result, its ringing suppression performance is weaker in relatively flat regions than in regions with severe ringing, leaving the overall suppression effect suboptimal. Taken together, these two representative approaches highlight a persistent trade-off: methods that aggressively suppress ringing often sacrifice detail preservation, while those that retain high-frequency details tend to exhibit insufficient suppression in certain regions.

In summary, existing methods for ringing artifact suppression fall into two main categories. The first focuses on isolating or down-weighting overexposed regions but is inherently limited by the fact that energy distortion propagates beyond these regions before reconstruction. The second directly targets ringing patterns but struggles to balance effective suppression with faithful detail preservation. Consequently, achieving a solution that fundamentally eliminates ringing while maintaining high image quality remains an open challenge.

### 2.2. Neural Network Methods in Image Reconstruction

In recent years, neural networks have been extensively studied in the field of image reconstruction, benefiting from their powerful nonlinear modeling capabilities and demonstrating superior performance over traditional model-based iterative algorithms. In 2020, Dun et al. proposed a Res-Unet-based reconstruction algorithm [18], which introduced residual computation modules into the U-Net framework to learn the blur residual between clear and blurred images, achieving relatively high reconstruction accuracy. This method employs spatially invariant convolution kernels and is thus suited for scenarios with shift-invariant PSFs. In 2021, Chen et al. extended this architecture by integrating a field-of-view attention mechanism and dynamic convolution modules, proposing the FOV-KPN dynamic convolutional neural network [19], which exhibits strong adaptability to imaging conditions with spatially varying PSFs. In 2022, the Transformer architecture was introduced into image restoration to overcome the limited receptive field of CNNs. Wang et al. proposed Uformer, a general U-shaped Transformer that builds a hierarchical encoder–decoder network using Transformer blocks [21]. It features a locally-enhanced window (LeWin) Transformer block that performs non-overlapping window-based self-attention to reduce computational complexity on high-resolution feature maps, along with a learnable multi-scale restoration modulator to adjust features across decoder layers. Zamir et al. developed Restoration Transformer (Restormer) [22], an efficient Transformer designed for high-resolution image restoration, which makes key modifications to multi-head attention and feed-forward networks to capture long-range pixel interactions while remaining computationally feasible for large images. It achieves state-of-the-art results on tasks including image deraining, motion deblurring, defocus deblurring, and image denoising. Chen et al. [23] proposed a simple yet effective baseline that exceeds prior state-of-the-art methods with improved computational efficiency. By revealing that nonlinear activation functions such as Sigmoid, ReLU, and GELU can be replaced by multiplication or removed, they derived a Nonlinear Activation Free Network (NAFNet) that achieves competitive performance on deblurring and denoising benchmarks with significantly reduced computational costs [23].

Building upon the FOV-KPN architecture, our research group recently proposed an image reconstruction algorithm that cascades non-blind deconvolution with a FOV-KPN neural network [20]. In this design, Wiener deconvolution is first applied to reduce blur, followed by feature extraction and image reconstruction via the FOV-KPN network, leading to improved restoration fidelity and enhanced robustness to spatially variant PSFs.

Despite the progress made by these neural network-based approaches, existing methods generally do not adequately account for ringing artifacts, resulting in degraded reconstruction quality under overexposed conditions. This limitation stems from the fact that saturated pixels severely violate the linear blur model assumptions, rendering their restoration dependent on highly nonlinear, content-adaptive priors that remain insufficiently addressed in current frameworks.

## 3. Proposed Method

**Figure 1:**
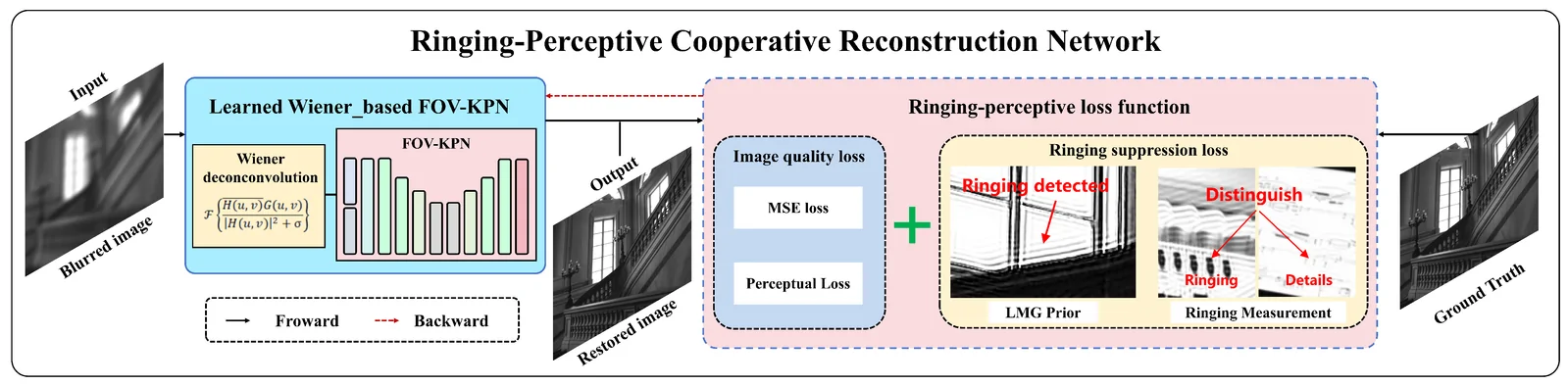
The proposed RPCR-Net. The proposed network performs deep image reconstruction using a learned Wiener FOV-KPN framework, incorporating a ringing-perceptive loss function to explicitly identify and constrain ringing data. Solid black arrows indicate the forward data flow, whereas the red dashed arrow denotes backward gradient propagation during training. The colored backgrounds are used to distinguish different functional modules and do not encode additional quantitative information. The green plus sign denotes the combination of the image quality loss and the ringing suppression loss, while the red annotation arrows highlight ringing artifacts and image-detail regions. This design facilitates the effective recognition and suppression of ringing artifacts while preserving the integrity of fine image details.

The proposed RPCR-Net comprises two key components: a learned Wiener-based FOV-KPN neural network previously developed by our team [20] and a ringing-perceptive loss function, as illustrated in Figure 1. The neural network is responsible for restoring image details from sensor measurements, while the ringing-perceptive loss function introduces a ringing-suppression regularization term to guide network optimization toward minimizing ringing artifacts. Further details are provided in Section S5 of the Supplementary Materials.

### 3.1. Ringing-Perceptive Loss Function

**Figure 2:**
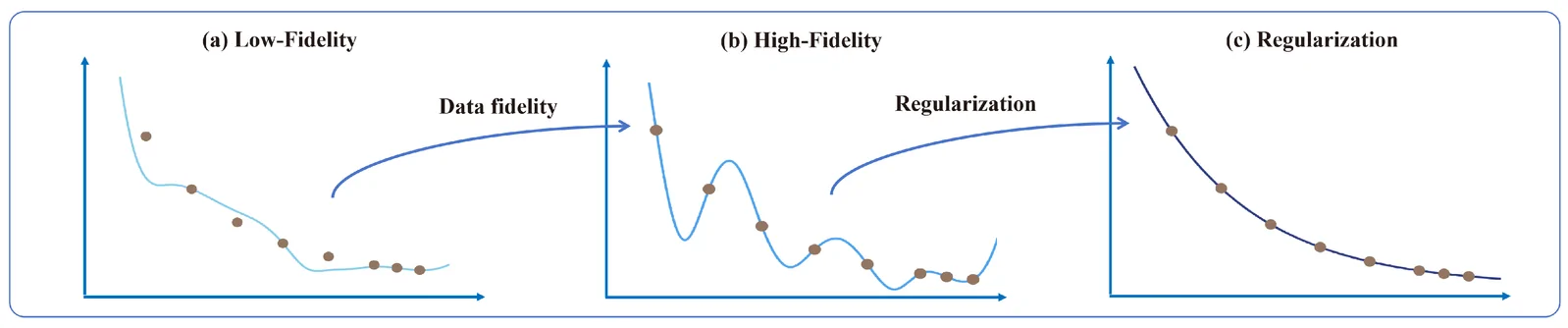
Effect of the loss function and regularization on neural network-based image restoration. The dots represent the data points used for model fitting, and the line represents the corresponding fitted model curve. (**a**) The loss function computes gradients with respect to optimization parameters and drives model convergence via gradient descent. (**b**) Overfitting may lead to oscillations in the reconstruction (e.g., ringing artifacts), even when the result closely matches the reference image. (**c**) Regularization constrains the reconstruction process to mitigate such oscillations, thereby producing more accurate results.

The loss function is central to guiding neural network training toward high restoration performance. In neural image restoration, the network computes the gradient of the loss with respect to the parameters and employs gradient descent for model convergence, as shown in Figure 2a. However, the optimization process is prone to overfitting. As illustrated in Figure 2b, while the reconstruction aligns well with the reference, it often introduces oscillations that manifest as ringing. Regularization mitigates these artifacts by constraining the reconstruction, yielding more accurate results (Figure 2c). This section therefore focuses on designing a loss function that preserves reconstruction fidelity while suppressing ringing.

In this work, we construct a composite loss function within a multi-task learning framework, denoted as $L_{total}$. The base loss combines Mean Squared Error (MSE) and perceptual losses to enforce pixel-level fidelity and preserve basic image information, where the MSE loss is denoted as $L_{MSE}$ and the perceptual loss as $L_{prec}$. Building upon this foundation, we introduce a ringing-perceptive loss that integrates the LMG prior and a ringing measurement term, leveraging their sensitivity to ringing artifacts. The LMG prior is denoted as $L_{lmg}$ and the ringing measurement term as $L_{ring}$. This design effectively suppresses ringing while maintaining fine image details. The overall loss function is defined as a weighted sum of these individual components:

(1) $$L_{total}=L_{MSE}+\lambda_{prec}L_{prec}+\lambda_{ring}\left(L_{ring}+\lambda_{lmg}L_{lmg}\right),$$

where $\lambda_{prec}$ represents the coefficient of the perceptual loss, $\lambda_{ring}$ is the overall coefficient of the ringing-perceptive loss, and $\lambda_{lmg}$ is the coefficient of the LMG prior.

#### 3.1.1. MSE Loss

The MSE Loss is a fundamental loss function in image restoration tasks, measuring the Euclidean distance in pixel space between the network output and the ground truth image [19]:

(2) $$L_{MSE}=\frac{1}{N}\sum_{i=1}^{N}{∥y_{i}-{\hat{y}}_{i}∥}_{2}^{2},$$

where *N* is the total number of pixels, $y_{i}$ is the value of the *i*-th pixel in the ground truth image, and ${\hat{y}}_{i}$ is the corresponding network prediction. The MSE Loss promotes an average optimal solution for all pixels, aiding the reconstruction of an image whose average data is closest to the original.

#### 3.1.2. Perceptual Loss

Perceptual Loss is based on the characteristics of the human visual system and optimizes visual quality by comparing differences in deep feature spaces. We use the VGG-19 network as the feature extractor, specifically utilizing the feature maps from the block2-conv2 layer [19]:

(3) $$L_{prec}=\frac{1}{C_{j}H_{j}W_{j}}{∥\phi_{j}\left(\hat{y}\right)-\phi_{j}\left(y\right)∥}_{2}^{2},$$

where $\phi_{j}$ represents the feature extractor for the *j*-th layer of the VGG-19 network. The advantage of perceptual loss lies in its focus on feature similarity rather than strict pixel-level matching, thereby enabling the generation of visually more natural results.

#### 3.1.3. Ringing-Perceptive Loss

The ringing prior loss comprises two complementary components: the LMG Prior model [16] and the Ringing Measurement model [17]. The LMG Prior regularization term operates by analyzing the gradient information distributed across the image, explicitly leveraging the strong intrinsic correlation between ringing artifacts and localized gradient anomalies in order to both identify and subsequently suppress regions contaminated by ringing. Building upon this foundation, the Ringing Measurement model provides an additional layer of discrimination: it distinguishes between genuine structural details residing in areas that naturally exhibit large gradient magnitudes and those gradient fluctuations that are artificially induced by ringing during the image reconstruction process. By analyzing the phenomenon of abnormally amplified gradient responses, this model ensures that image details are preserved with high fidelity, complementing the ringing removal capabilities already established by the LMG Prior. Together, these two models collaborate to achieve an effective trade-off between detailed image reconstruction and robust ringing suppression.

hlLMG Prior Regularization Term. The LMG prior serves to regularize the reconstruction process by penalizing gradient anomalies that are characteristically associated with the presence of ringing artifacts [16]. More specifically, the ringing phenomenon introduces oscillatory intensity patterns that manifest as abnormally elevated gradient magnitudes within confined local regions. In contrast to natural image edges, which typically give rise to a single, well-defined gradient peak, ringing regions tend to generate multiple high-gradient peaks clustered in close spatial proximity. Within the local maximum gradient framework, these artifact-induced peaks dominate the gradient landscape, resulting in distinctively elevated LMG values that function as reliable and readily detectable indicators of ringing contamination. Consequently, the minimization of the LMG loss term effectively discourages the formation of such irregular gradient structures, thereby suppressing the visibility and impact of ringing artifacts.

The mathematical definition of LMG is as follows. For a given pixel position *x*, we first define its neighborhood P(x), typically using a local window of size 3×3 or 5×5. Within this neighborhood, the gradient magnitude is computed, and the maximum value is taken as the LMG value for that position. The gradient is calculated using the central difference method: $\nabla_{h}I\left(i,j\right)=I\left(i,j+1\right)-I\left(i,j-1\right)$, $\nabla_{v}I\left(i,j\right)=I\left(i+1,j\right)-I\left(i-1,j\right)$. The gradient magnitude is:

(4) $$\left|\nabla I\right|=\sqrt{{\left(\nabla_{h}I\right)}^{2}+{\left(\nabla_{v}I\right)}^{2}},$$

This discrete difference approximation can effectively capture the local variation intensity of an image. For the reconstructed image $\hat{x}$, we compute the maximum |∇I| value within the neighborhood P(x) of each pixel, which is defined as the LMG, denoted as:

(5) $$\mathrm{LMG}\left(\hat{x}\right)=\max\left(\max\left(|\nabla I(\hat{x})|\right)\right).$$

In the loss function, this model is expressed as:

(6) $$L_{\mathrm{lmg}}=\frac{1}{N}\sum_{p}{\left(\mathrm{LMG}{\left(\hat{y}\right)}_{p}-\mathrm{LMG}{\left(y\right)}_{p}\right)}^{2},$$

where *N* is the number of pixels, $\mathrm{LMG}(\hat{x})$ and LMG(y) denote the local maximum gradient maps of the predicted image and the ground-truth clear image, respectively.

**Figure 3:**
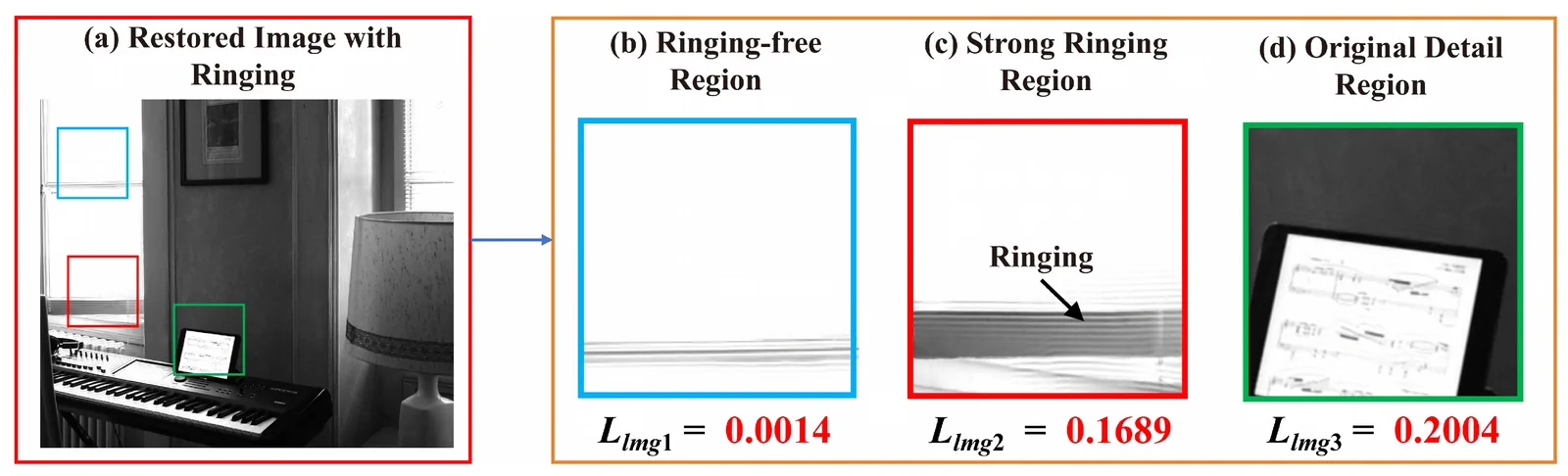
LMG responses in different image regions and the limitation of ringing localization. (**a**) An image with evident ringing artifacts. (**b**) In neural network reconstruction results without ringing optimization, LMG values are low in regions without prominent ringing. (**c**) High LMG values appear in regions with significant ringing, demonstrating the mechanism’s ability to localize ringing artifacts. (**d**) However, high LMG values also occur in texture-rich areas with genuine structures, indicating that the mechanism cannot reliably distinguish ringing from real image details, which may lead to over-suppression of fine textures.

As illustrated in Figure 3, the image presented in Figure 3a exhibits conspicuous and clearly discernible ringing artifacts. In the neural network reconstruction results obtained without any dedicated ringing optimization mechanism, the computed LMG values remain comparatively low within image regions where ringing artifacts are either absent or visually inconspicuous, as demonstrated in Figure 3b. In stark contrast, a pronounced and strong response emerges precisely within those spatial regions where significant ringing artifacts are present, as shown in Figure 3c. This empirical observation confirms that the mechanism is indeed capable of accurately localizing ringing artifacts and holds considerable potential for deployment as a dedicated regularization term to guide the process of ringing suppression. It should be noted, however, that elevated LMG values are not exclusively confined to artifact-laden regions; they also appear with notable frequency in texture-rich areas that contain authentic and structurally meaningful image details, as exemplified in Figure 3d. This phenomenon exposes a clear and inherent limitation of the mechanism: owing to its fundamental inability to reliably discriminate whether a given high LMG response originates from genuine ringing artifacts or from legitimate high-contrast image structures, the model exhibits an undesirable tendency to misclassify normal, high-contrast features as ringing and to subsequently suppress them. As a direct consequence, this misclassification leads to an irreversible loss of fine image detail and an attendant degradation in overall reconstruction quality.

Ringing Measurement Regularization Term. Ringing artifacts manifest as mid-frequency oscillations that mix with genuine image structures, making their detection particularly challenging, especially in textured regions. To regularize the reconstruction, we enforce structural consistency between the blurred input image b and the deblurred result $\hat{x}$ [17]. In the absence of artifacts, the two images should share similar gradient distributions; ringing introduces unnatural spatial oscillations that disproportionately amplify the gradient response of $\hat{x}$ relative to b, thereby deviating from the expected natural profile.

To reliably capture these artifacts while ignoring noise and fine details, we adopt the detail-robust derivative operators proposed in the literature. These kernels are designed to extract high-frequency variations along specific directions while suppressing low-amplitude textures. The horizontal and vertical operators are defined as [17]:

(7) $$H_{h}=\frac{1}{32}\left[\begin{array}{ccccc} -1 & -2 & 0 & 2 & 1\\ -2 & -4 & 0 & 4 & 2\\ -1 & -2 & 0 & 2 & 1 \end{array}\right],\;H_{v}=\frac{1}{32}\left[\begin{array}{ccc} -1 & -2 & -1\\ -2 & -4 & -2\\ 0 & 0 & 0\\ 2 & 4 & 2\\ 1 & 2 & 1 \end{array}\right].$$

Their directional specificity matches the oscillatory nature of ringing, enhancing artifact-induced gradients in the horizontal and vertical directions and facilitating subsequent detection.

In a valid reconstruction, ringing artifacts are expected to produce stronger directional gradient magnitudes in $\hat{x}$ than in b. Conversely, true sharp edges that are already present in the blurred image should not be penalized as ringing. We therefore define a gradient response difference that retains only positive deviations [17]:

(8) $$\delta ={\left(|H_{h}\hat{x}\left|-\right|H_{h}b|\right)}_{+}\;+\;{\left(|H_{v}\hat{x}\left|-\right|H_{v}b|\right)}_{+},$$

where |·| denotes element-wise absolute value and ${\left(\cdot \right)}_{+}=\max\left(\cdot ,0\right)$ sets negative entries to zero. This non-negative truncation guarantees that regions where the blurred image already exhibits strong gradients are not mistakenly counted as ringing, focusing the measure exclusively on artifact-induced overshoots.

The resulting regularization term is expressed as the $\ell_{1}$ norm of δ:

(9) $$L_{ring}={∥\delta ∥}_{1}.$$

The $\ell_{1}$ norm is employed here because of its high-peak-preserving property: it penalizes the overall presence of ringing without aggressively smoothing the few large responses that may arise near genuine strong edges. Minimizing $L_{ring}$ during deblurring thus suppresses ringing artifacts while respecting the original image structures.

In the reconstructed image, areas containing genuine details have gradient distributions similar to the original image, while ringing regions exhibit significant differences. The differential calculation described above helps avoid misclassifying high-gradient values from real details as ringing and suppressing them. Thus, it allows for the removal of artifacts while preserving the original image information. However, due to its gradient amplification characteristic, this method produces a disproportionately high response to strong ringing while providing a relatively insufficient response to weaker ringing. Consequently, although it can effectively detect severe ringing, its ability to suppress mild ringing is limited.

**Figure 4:**
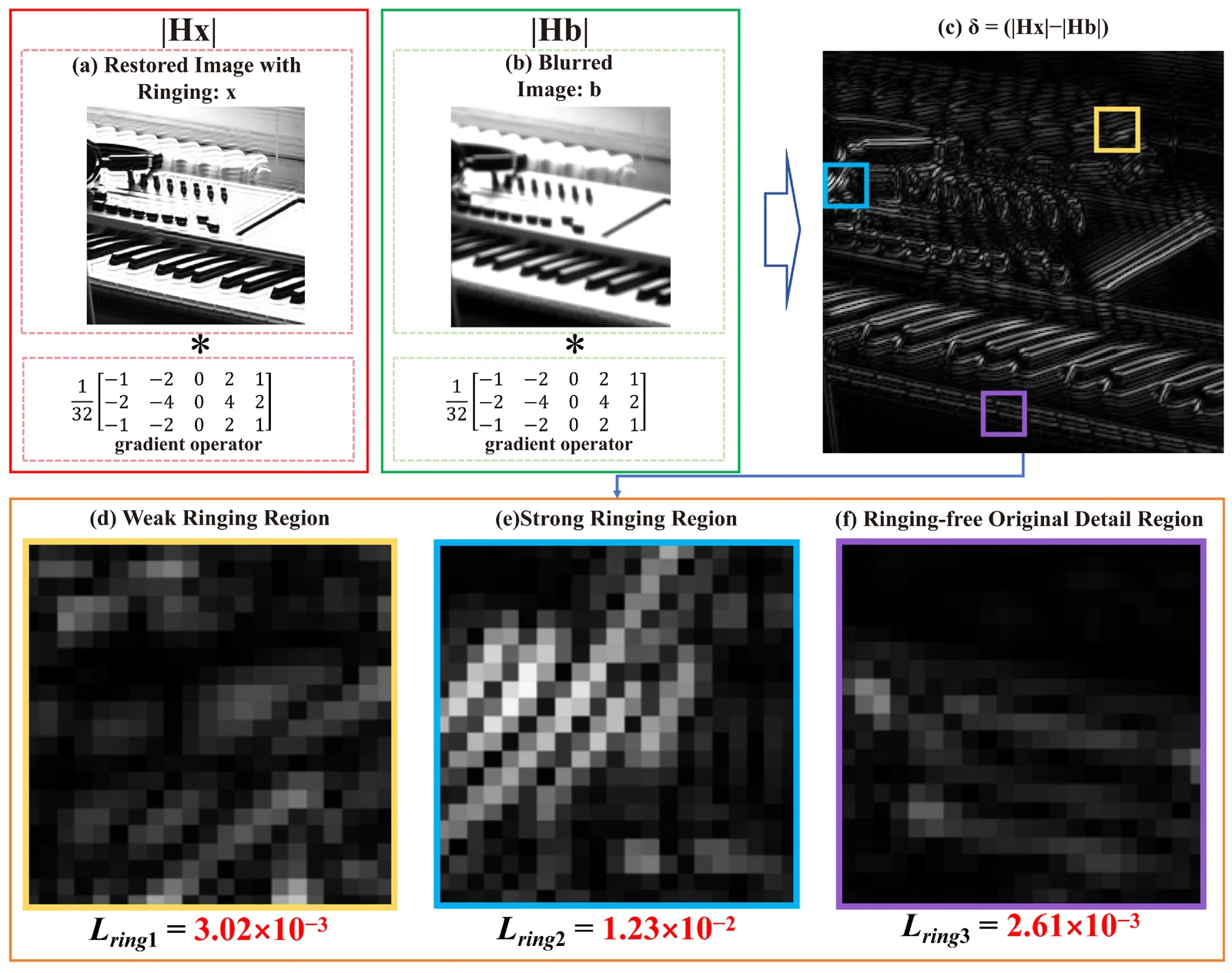
Ringing Measurement Regularization in different image regions and the limitation of weak ringing identification. (**a**) Neural network reconstructed image without ringing optimization; (**b**) Original image; (**c**) δ map computed between (**a**,**b**); (**d**) δ value in a weak ringing region; (**e**) δ value in a strong ringing region (large); (**f**) δ value in a non-ringing region containing original details.

To verify this conclusion, we calculated the ringing measurement value δ between the neural network reconstructed image without ringing optimization (denoted as Figure 4a) and the original image (denoted as Figure 4b), as shown in Figure 4c. As illustrated in Figure 4e, the δ value is $1.23\times 10^{-2}$ in regions with prominent ringing, while it remains relatively low in non-ringing areas that contain original image details (e.g., Figure 4f), where the value is $2.61\times 10^{-3}$. This indicates that the reconstruction process effectively avoids misclassifying genuine image details as ringing, thereby preserving real details reasonably well. However, as shown in Figure 4d, the δ value in weak ringing regions is $3.02\times 10^{-3}$, which is considerably smaller than that in strong ringing regions. This suggests that the method has low sensitivity in detecting mild ringing artifacts. Consequently, during the gradient amplification process, this ringing measurement method exhibits insufficient sensitivity for suppressing non-severe ringing, which may lead to inadequate correction of ringing artifacts.

Based on the analysis above, this study integrates the ringing measurement regularization term and the LMG prior to construct a collaborative suppression mechanism. Within this mechanism, the LMG prior efficiently suppresses ringing by globally identifying and penalizing high-gradient extremes. Concurrently, the ringing measurement term accurately distinguishes ringing from genuine details by comparing the gradient similarity between the reconstructed and the blurred images using specialized gradient operators, thereby preserving the original image structure. This combined strategy effectively suppresses ringing artifacts while enhancing the ability to retain original details, thereby comprehensively improving image restoration quality.

### 3.2. The Learned Wiener-Based FOV-KPN Neural Network

The learned Wiener-based FOV-KPN neural network is composed of a Wiener deconvolution cascaded with an FOV-KPN neural network [19,20]. Wiener deconvolution restores images in the frequency domain using the fast Fourier transform [24,25]. However, it often yields compromised image quality due to amplified noise and the introduction of artifacts. The subsequent FOV-KPN further restores image details through three core components: the FOV Block, the Deformable ResBlock, and the KPN Block. Specifically, the FOV Block first extracts multi-scale features from the input image, which are then refined by the Deformable ResBlock to model non-rigid spatial deformations. Finally, the KPN Block predicts pixel-wise adaptive kernels to generate the restored image with enhanced details and reduced artifacts.

## 4. Experiments and Results

### 4.1. Experimental Setups

To validate the proposed method, we employed an off-axis three-mirror freeform imaging system developed by our team, with a field of view of 7.8°×6.5°, an aperture of F/2.8, and a spectral range of 400–1050nm. Using this system, we generated overexposed image datasets for training purposes. The dataset construction process consisted of the following steps:

1. Normalize the 16-bit image and convert it to double-precision floating-point format, obtaining an image *I* with pixel values within the range [0,1];
2. Retrieve the maximum value of the image, denoted as $I_{\max}$;
3. Scale the image: $I_{1}=I/I_{\max}$, so that its maximum value is mapped to 1;
4. Select a scaling factor σ greater than 1 and further amplify the image: $I_{2}=I_{1}\times \sigma$, thereby generating an image where some pixel values exceed 1 to simulate overexposure;
5. Convolve the image $I_{2}$ with the optical system’s PSFs to obtain the blurred image $I_{3}$;
6. Add pixel-wise Gaussian–Poisson noise to $I_{3}$ to simulate realistic sensor noise;
7. Truncate all pixel values in $I_{2}$ and $I_{3}$ that are greater than 1 to 1, resulting in $I_{2}^{\prime }$ and $I_{3}^{\prime }$, simulating the detector’s truncation of overexposed pixels;
8. Pair the overexposed clear image $I_{2}^{\prime }$ with the overexposed blurred image $I_{3}^{\prime }$ to form an image pair for training and testing.

**Figure 5:**
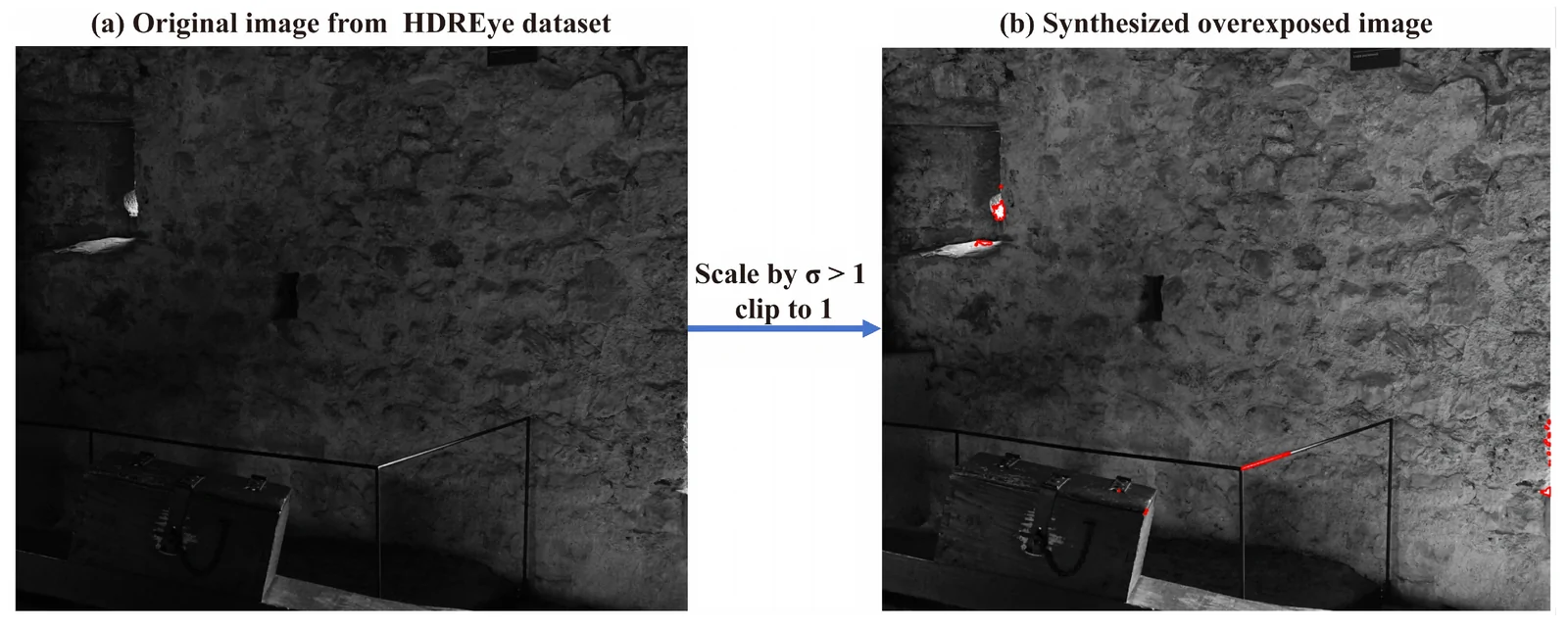
Illustration of dataset construction. (**a**) Original high dynamic range image (pixel values normalized to [0, 1], full dynamic range retained); (**b**) Synthesized overexposed image (amplified by a scaling factor σ > 1 and then clipped to 1 to simulate detector saturation). The red region in (**b**) indicates overexposed pixels.

Following the above procedure, we constructed a dataset for training an overexposure restoration neural network. Using images from the HDREye dataset—which were first partitioned into training and test splits at the image level to avoid any scene overlap—we cropped different regions and applied various scaling factors σ to generate overexposed samples; further details are provided in Section S2 of the Supplementary Materials. An example of this process is shown in Figure 5. The resulting dataset consists of 200 high-quality images covering diverse scenes and varying degrees of overexposure. Among them, 100 are normally exposed images with ideal lighting conditions and full dynamic range, while the other 100 exhibit different levels of overexposure. This balanced dataset design aims to enable the model to learn both regular image features and effectively optimize against the ringing artifacts caused by overexposure. The overexposure level for each image was controlled by a randomly chosen σ from [1.1, 1.4], ensuring a diverse range of saturation degrees. All images were uniformly resized to a resolution of 1216 × 1024 pixels. Subsequently, we selected 50 normally exposed images and 50 overexposed images to form the training dataset, while the remaining 50 normally exposed images and 50 overexposed images constituted the test dataset.

The sensor noise injected in Step 6 follows a pixel-wise Gaussian–Poisson noise model:

(10) $$g\left(x,y\right)=\eta_{p}\left[I_{3}\left(x,y\right);\sigma_{p}\right]+\eta_{g}\left[I_{3}\left(x,y\right);\sigma_{g}\right],$$

where $\eta_{p}\left(\cdot ;\sigma_{p}\right)$ and $\eta_{g}\left(\cdot ;\sigma_{g}\right)$ represent the Poisson and Gaussian noise components, respectively. The Poisson term models photon shot noise, while the Gaussian term accounts for read noise, dark current, and other electronic noise sources. The noise parameters were calibrated from our experimental prototype following the procedure described in Ref. [20], yielding $\sigma_{g}=1.1\times 10^{-3}$ and $\sigma_{p}=8.8\times 10^{-5}$. Further details are provided in Section S1 of the Supplementary Materials.

To train the learned Wiener-based FOV-KPN neural network, we followed the parameter settings in Ref. [20]. Here, we focus on the parameter configuration of the loss function. To balance the influence of each regularization loss on the training process, we adopted common training strategies, aiming to keep their numerical scales roughly consistent. By monitoring the values of each loss $L_{MSE}$, $L_{prec}$, $L_{ring}$, $L_{lmg}$ during training, we used $L_{MSE}=1$ as the benchmark and determined the corresponding scaling coefficients as follows: $\lambda_{prec}=0.005$, $\lambda_{ring}=5\times 10^{-8}$, $\lambda_{lmg}=2\times 10^{6}$. Further details are provided in Section S3 of the Supplementary Materials. This setting ensures that each regularization term contributes comparably during training, thereby maintaining a balanced overall loss.

### 4.2. Ablation Study

**Figure 6:**
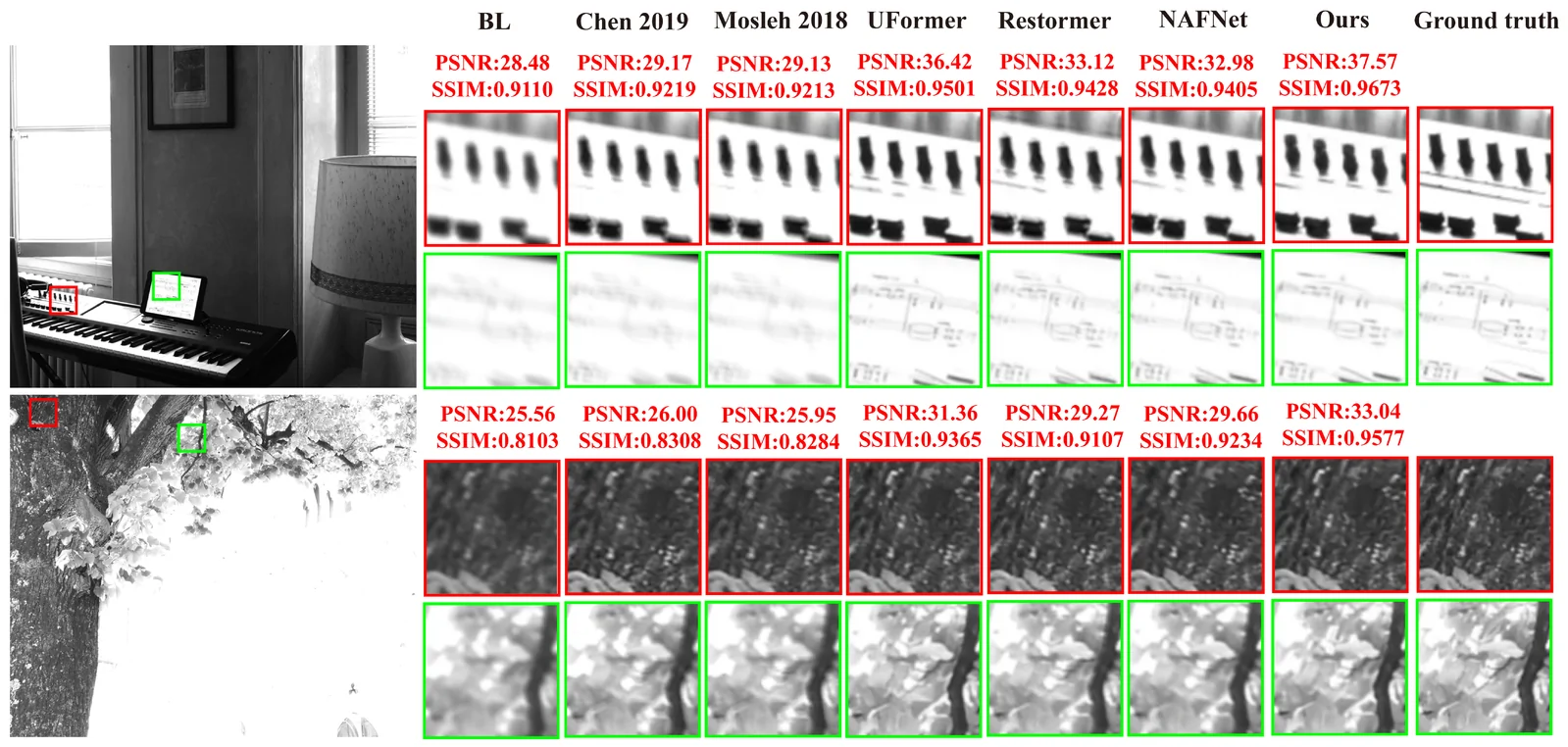
Comparison of simulation results between our method and traditional methods. Reconstruction performance, including PSNR (dB) and SSIM, is evaluated for six methods: the LMG Prior method proposed by Chen et al. [16], the Ringing Measurement method proposed by Mosleh et al. [17], Uformer [21], Restormer [22], NAFNet [23], and our proposed RPCR-Net. The reconstruction result of our proposed method is presented on the left side, while magnified results from different methods are displayed on the right side, with corresponding positions highlighted on the left side.

**Figure 7:**
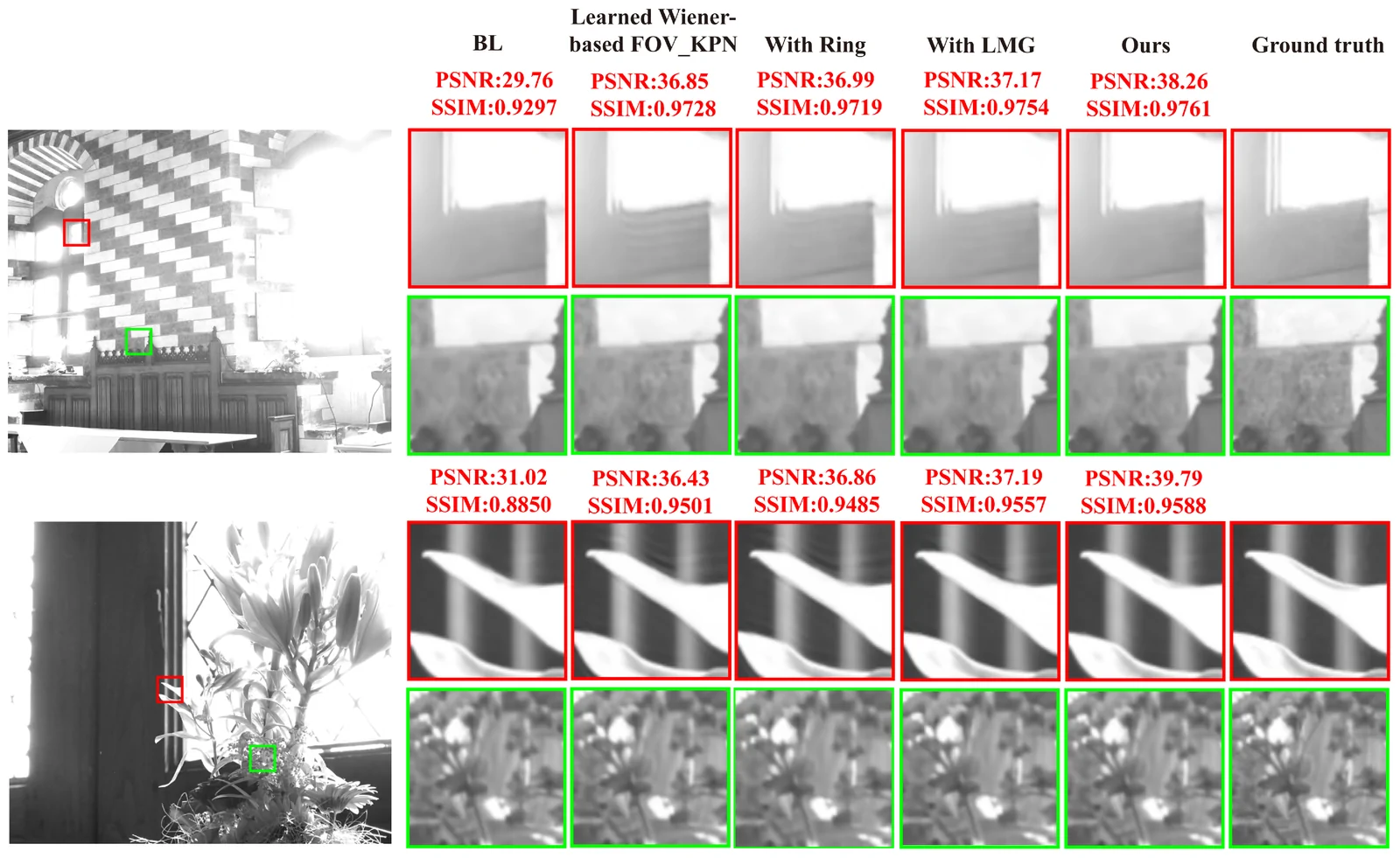
Ablation study. Reconstruction performance, including PSNR (dB) and SSIM, is evaluated for four configurations: the learned Wiener-based FOV-KPN, the learned Wiener-based FOV-KPN with the ringing measurement term, the learned Wiener-based FOV-KPN with the LMG prior, and our proposed RPCR-Net. The reconstruction result of our proposed method is presented on the left side, while magnified results from different methods are displayed on the right side, with corresponding positions highlighted on the left side.

To verify the performance advantages of the proposed RPCR-Net, we first compared it with traditional iterative algorithms and recent deep learning methods, followed by validation of the core components of the framework. The framework incorporates two key regularization modules: the ringing measurement regularization term and the LMG prior regularization term. The qualitative and quantitative results are presented in Table 1 and Table 2, Figure 6 and Figure 7, respectively.

As shown in Table 1, the traditional approaches—LMG Prior by Chen et al. and Ringing Measurement by Mosleh et al.—effectively suppress strong ringing artifacts, but their recovered images suffer from a noticeable loss of fine details. In contrast, the generic deep learning methods (NAFNet, Uformer, Restormer, and the learned Wiener-based FOV-KPN) are able to preserve more image structures, yet they leave obvious ringing residuals in overexposed regions, indicating weak artifact suppression. Uformer achieves a relatively high PSNR of 36.95 dB, which is better than the learned Wiener-based FOV-KPN, but still falls behind our method. The reason we build our network upon the learned Wiener-based FOV-KPN backbone rather than Uformer is that the learned Wiener-based FOV-KPN is a lightweight backbone with a small model size and fast inference. As shown in Table 1, its inference time and number of parameters are significantly smaller than those of NAFNet, Uformer, and Restormer, making it a more efficient choice for practical deployment. Our proposed RPCR-Net, built upon this backbone and equipped with the synergistic ringing-perceptive loss, achieves the highest PSNR of 37.06 dB and SSIM of 0.9549, significantly outperforming all compared methods in both artifact suppression and detail preservation. The visual comparisons in Figure 6 confirm that RPCR-Net removes ringing more thoroughly while restoring fine textures better than other techniques.

To further quantify perceptual quality and statistical reliability, we computed LPIPS (Learned Perceptual Image Patch Similarity), NIQE (Natural Image Quality Evaluator), and the standard deviations of all metrics; the full results are reported in Section S7 of the Supplementary Materials. Our method consistently delivers the best perceptual scores and the smallest variance, indicating robust performance across diverse test images.

Subsequently, we conducted ablation studies. The baseline learned Wiener-based FOV-KPN network, which is not designed for overexposed scenes, yields a PSNR of 34.76 dB and an SSIM of 0.9459 (Table 2). As illustrated in Figure 7, severe ringing appears in saturated areas, degrading the overall reconstruction. Adding the ringing measurement term alone improves the PSNR by 1.32 dB and SSIM by 0.0012; strong ringing is largely removed, though weak artifacts persist. Introducing the LMG prior alone results in an increase of 1.59 dB in PSNR and 0.0023 in SSIM over the baseline; ringing is suppressed, but with a modest sacrifice in fine detail preservation. The proposed RPCR-Net jointly incorporates both terms within a unified framework, achieving the highest PSNR and SSIM values—outperforming the baseline by 2.30 dB in PSNR and 0.0090 in SSIM. In addition to superior quantitative metrics, the proposed approach demonstrates a pronounced capability to suppress ringing artifacts while effectively preserving fine image structures and textural details, thereby contributing to a substantial overall enhancement in perceptual reconstruction quality. Furthermore, we verify that after adding the ringing-perceptive loss, the inference time and parameter count of the network remain identical to those of the baseline, confirming that this improvement does not degrade the network’s computational efficiency. Further details are provided in Sections S6 and S7 of the Supplementary Materials.

### 4.3. Restoration Results on Captured Images

**Figure 8:**
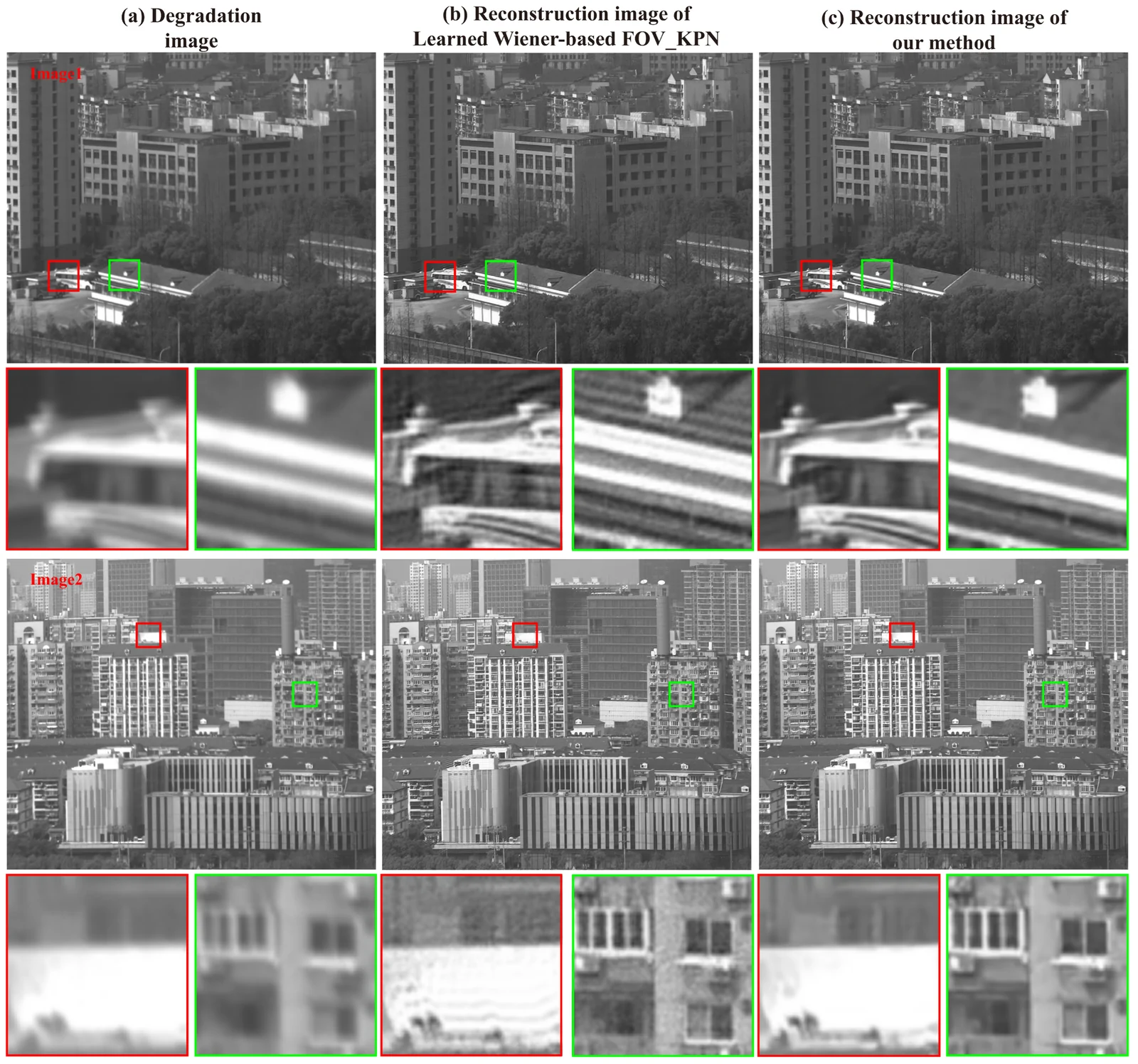
Real-world imaging performance comparison on a prototype-captured scene. (**a**) Raw image acquired under natural illumination, featuring overexposed regions typical of high-dynamic-range scenes. (**b**) Restoration result from the baseline Learned Wiener-based FOV-KPN, which does not explicitly model ringing artifacts. Noticeable ringing artifacts are visible along high-contrast edges. (**c**) Restoration result using our proposed RPCR-Net. Ringing artifacts are effectively suppressed while fine image details are preserved.

To experimentally validate the effectiveness of the proposed method in a real-world environment, we conducted outdoor experiments in the Siping Road campus of Tongji University. Figure 8a shows a raw image acquired by the prototype under natural illumination, which includes overexposed regions typical of high-dynamic-range scenes. For comparison, we performed restoration using both the baseline Learned Wiener-based FOV-KPN network—which does not explicitly model ringing artifacts—and our proposed RPCR-Net. The corresponding results are shown in Figure 8b,c, respectively.

As observed, the baseline method struggles in overexposed areas, producing noticeable ringing artifacts and oscillatory patterns along high-contrast edges, which degrade visual quality. In contrast, our method effectively suppresses these artifacts while preserving fine image details. The reconstructed images exhibit clean, natural contours without obvious streaking or distortion, demonstrating that the proposed ringing-perceptive regularization generalizes well to real captured data. These results confirm that the combination of precise hardware fabrication and artifact-aware neural reconstruction enables high-quality imaging in practical settings, highlighting the practical utility and robustness of our approach. More results are provided in Section S4 of the Supplementary Materials.

## 5. Discussion

### 5.1. Interpretation of Key Results

Our ablation study confirms the effectiveness of the core design, demonstrating that both proposed regularization components contribute substantially to performance improvement. The LMG prior proves particularly effective in suppressing ringing artifacts, while the ringing measurement is designed to eliminate ringing without sacrificing image details. Their synergistic combination yields the best performance, achieving a PSNR of 37.06 dB and an SSIM of 0.9549, and striking an optimal balance between ringing suppression and detail preservation. Furthermore, we validate the practical utility of our method on real-world data. Unlike the learned Wiener-based FOV-KPN network, which does not model ringing, our method effectively suppresses ringing artifacts in typical overexposed regions while maintaining high-fidelity detail reconstruction.

### 5.2. Comparison with Conventional Methods

Experimental results demonstrate that our method significantly outperforms conventional reconstruction algorithms. Relative to traditional iterative optimization-based image reconstruction algorithms, our method exhibits stronger detail discrimination. Specifically, it improves the PSNR by 7.07 dB and the SSIM by 0.0595 over the method of Mosleh et al., and by 7.00 dB and 0.0584 over that of Chen et al. Compared with the baseline learned Wiener-based FOV-KPN, which is not optimized for overexposed scenes, our full method incorporates a ringing suppression regularization term. This term effectively reduces ringing artifacts in overexposed scenes while preserving detail discrimination, leading to PSNR and SSIM gains of 2.30 dB and 0.0090, respectively. Visually, ringing artifacts are greatly reduced, producing restored images with sharper and more natural contours.

### 5.3. Limitations and Further Analysis

Despite its clear advantages in detail recovery and ringing suppression, our algorithm has several limitations that warrant further optimization. The inclusion of complex network structures—such as Wiener deconvolution, FOV blocks, dynamic convolution blocks, and KPN blocks—incurs a higher computational load. This leads to prolonged processing times on resource-constrained edge hardware platforms, thereby limiting the real-time imaging capability of the system. Consequently, deploying the method on edge devices with strict real-time requirements calls for additional optimization to improve operational efficiency.

### 5.4. Future Work Directions

Building on the achievements of this study, future work will focus on improving real-time imaging performance. By exploring model lightweighting techniques—including network pruning, quantization, and knowledge distillation—we aim to reduce neural network redundancy while preserving imaging quality and ringing suppression effectiveness. These efforts are expected to enhance real-time performance and facilitate engineering deployment, ultimately enabling our framework to run on resource-constrained embedded platforms or real-time imaging systems.

## 6. Conclusions

In summary, we proposed RPCR-Net to suppress ringing artifacts in overexposed image reconstruction. Built upon a learned Wiener-based FOV-KPN, it integrates a ringing-perceptive mechanism that combines an LMG prior with a multi-scale ringing measurement model. This design preserves image details while effectively identifying and suppressing anomalous gradient oscillations caused by saturation. Experiments show that RPCR-Net improves PSNR from 34.76 dB to 37.06 dB and SSIM from 0.9459 to 0.9549 over the baseline, significantly outperforming existing methods. Real-world captured results further demonstrate effective ringing suppression along edges and in strongly saturated regions, yielding sharper contours and enhanced perceptual quality. Collectively, these results establish RPCR-Net as a robust post-processing solution for remote sensing applications.

## Figures and Tables

**Table 1:** Comparison of our proposed method with iterative algorithms and recent deep learning methods in terms of reconstruction quality, model complexity, and inference efficiency. The best results are highlighted in bold. PSNR (dB) and SSIM are reported as mean values over the test set. LMG Prior and Ringing Measurement are traditional iterative algorithms without learnable parameters or GPU inference time. The best results are highlighted in bold.

Method	PSNR (dB)	SSIM	Parameters (M)	Inference Time (ms)
Blurred	29.08	0.8795	/	/
LMG Prior (Chen et al.) [16]	30.06	0.8965	1.1	53.1
Ringing Measurement (Mosleh et al.) [17]	29.99	0.8954	1.1	66.8
NAFNet [23]	30.67	0.8987	67.8	45.2
Uformer [21]	36.95	0.9501	20.6	72.1
Restormer [22]	32.17	0.9500	26.1	58.3
Learned Wiener-based FOV-KPN [20]	34.76	0.9459	4.0	28.4
**RPCR-Net (Ours)**	**37.06**	**0.9549**	**4.0**	**28.4**

**Table 2:** Ablation study on loss components. Our proposed method achieves the highest PSNR and SSIM by combining Ring Loss and LMG Loss. “With Ring” indicates that only the ringing measurement loss is added, and “With LMG” indicates that only the LMG loss is added. Values are reported as means over the test set. The best results are highlighted in bold.

Method	Ring Loss	LMG Loss	PSNR (dB)	SSIM
Blurred	×	×	29.08	0.8795
Learned Wiener-based FOV-KPN	×	×	34.76	0.9459
With Ring	✓	×	36.08	0.9471
With LMG	×	✓	36.35	0.9482
**Ours (RPCR-Net)**	✓	✓	**37.06**	**0.9549**

## Data Availability

The dataset and source code used in this study are publicly available at https://github.com/Ydh-wshxyz/A-Deep-Reconstruction-Framework-with-Ringing-Artifact-Suppression-for-Overexposed-Image (accessed on 23 August 2026). The original HDREye dataset is available at https://www.epfl.ch/labs/mmspg/downloads/hdr-eye (accessed on 23 August 2026).

## Supplementary Materials

The following supporting information can be downloaded at https://www.mdpi.com/article/10.3390/jimaging12090403/s1: Figure S1: Real-world imaging results on three additional outdoor scenes; Table S1: Raw magnitude of each loss term (10-epoch average); Table S2: Sensitivity analysis of the ringing measurement loss coefficient λ_*ring*_ (base value: 5 × 10^−8^); Table S3: Sensitivity analysis of the LMG loss coefficient λ_*lmg*_ (base value: 2 × 10^6^); Algorithm S1: Training Step of RPCR-Net; Table S4: Quantitative results on the external Flickr2K test set (30 images); Table S5: Full comparison including LPIPS, NIQE, and standard deviations; Table S6: Ablation study with LPIPS, NIQE, and standard deviations.

## Abbreviations

The following abbreviations are used in this manuscript:

FOV-KPN	Field-of-View shared Kernel Prediction Network
RPCR-Net	Ringing-perceptive Cooperative Reconstruction Network
LMG	Local Maximum Gradient
MSE	Mean Squared Error
PSNR	Peak Signal-to-Noise Ratio
SSIM	Structural Similarity Index
PSF	Point Spread Function
BL	Blurred Image
Uformer	U-shaped Transformer
Restormer	Restoration Transformer
NAFNet	Nonlinear Activation Free Network
LPIPS	Learned Perceptual Image Patch Similarity
NIQE	Natural Image Quality Evaluator
