# Supplementary material for: A Deep Reconstruction Framework with Ringing Artifact Suppression for Overexposed Remote Sensing Image Restoration
Source: J Imaging. 2026 Aug 26;12(9):403. doi: 10.3390/jimaging12090403 (PMC13608224; doi:10.3390/jimaging12090403)
Supplement: Supplementary file 1 [file jimaging-12-00403-s001.zip › jimaging-4314042-supplementary.pdf]

A Deep Reconstruction Framework with Ringing Artifact Suppression for Overexposed Remote Sensing Image Restoration: Supplementary Materials

This document provides supplementary information to "A Deep Reconstruction Framework with Ringing Artifact Suppression for Overexposed Remote Sensing Image Restoration". It includes detailed information on (1) Detector noise calibration, (2) Data split strategy and inter-set similarity analysis, (3) Loss weight determination and sensitivity analysis, (4) Additional real-world validation scenes, (5) Pseudocode of the RPCR-Net training procedure, (6) Generalization validation on an external dataset, (7) Perceptual quality and statistical analysis, and (8) Data availability.

S1. Detector Noise Calibration

To faithfully simulate the noise characteristics of real imaging sensors in our synthetic overexposed dataset, we adopted a pixel-wise Gaussian–Poisson noise model. The noise parameters were calibrated from the experimental prototype used in this study, following the laboratory-based measurement procedure detailed in Section 4.5 of the Supplemental Document of Ref. [20] of the main manuscript. The calibration platform consisted of an integrating sphere light source and the detector under test (FLIR BFS-U3-51S5M-BD2 CMOS detector). A tungsten halogen lamp produced uniform illumination, and the detector continuously captured 100 frames at each of several light intensity levels (adjusted via an aperture). The root-mean-square (RMS) noise at each pixel was computed across frames, and the squared noise was plotted against the normalized light intensity. A linear fit yielded the Poisson noise component (signal-dependent, slope) and the Gaussian noise component (signal-independent, square root of the intercept). The resulting calibrated parameters are $\sigma_g = 1.1 \times 10^{-3}$ for the Gaussian component and $\sigma_p = 8.8 \times 10^{-5}$ for the Poisson component. These values were used throughout the synthetic data generation pipeline described in Section 4.1 of the main manus.cript.

S2. Data Split Strategy and Inter-Set Similarity Analysis

A rigorous data split is critical for evaluating the generalization capability of deep learning models.In this study, we adopted an **image-level split** strategy to prevent any potential data leakage between the training and test sets. The HDREye dataset contains multiple independent source images covering diverse real-world scenes.We first randomly partitioned these source images into training and test sets at the image level.Only after this partition did we crop regions and apply scaling factors to generate the individual training and test samples.Consequently, all samples derived from a given source image belong exclusively to either the training set or the test set, ensuring zero content overlap at the parent-image level.

To quantitatively verify the absence of abnormal content similarity between the two sets, we computed the pairwise Structural Similarity Index Measure (SSIM) between all training and test images. The average inter-set SSIM was **0.3720** $\pm$ **0.1809**. These values are consistent with the natural variation expected between unrelated natural images, confirming that the image-level split effectively eliminates content overlap.

S3. Loss Weight Determination and Sensitivity Analysis

The composite loss function of RPCR-Net (Eq. (1) in the main manuscript) involves four weighting coefficients: λ_{prec}, λ_{ring}, and λ_{lmg} (with the MSE loss coefficient fixed at 1 as the reference). Improperly chosen

weights can cause one loss term to dominate the optimization, rendering the other terms ineffective. To avoid this, we adopted a principled **magnitude-balancing strategy** rather than arbitrary manual tuning.

First, we trained the network with all weights set to 1 for 10 epochs and recorded the raw magnitude of each loss term. The average values over these 10 epochs were:

Table S1: Raw magnitude of each loss term (10-epoch average).

**Loss Term**	**Raw Magnitude**
MSE loss	0.00144
Perceptual loss	0.31933
Ringing measurement loss	32344.67
LMG loss	0.01615

As shown, the ringing measurement loss is several orders of magnitude larger than the other terms. Using the MSE loss as the reference (coefficient = 1), we set the remaining coefficients to bring all terms to comparable magnitudes:

$$\lambda_{prec} = 0.00144/0.31933 \approx 0.0045 \to 0.005$$

$$\lambda_{ring} = 0.00144/32344.67 \approx 4.5 \times 10^{-8} \to 5 \times 10^{-8}$$

$$\lambda_{lmg} = (0.00144/0.01615)/\lambda_{ring} \approx 0.089/(5 \times 10^{-8}) \to 2 \times 10^{6}$$

This ensures that each loss term contributes with comparable magnitude at the beginning of training, preventing any single term from dominating the gradient updates.

To validate that the chosen coefficients are near-optimal, we conducted a **sensitivity analysis** by individually varying λ_{ring} and λ_{lmg} around their base values while keeping all other settings fixed. The experiments were performed using the "With Ring" and "With LMG" ablation configurations, respectively. The results are summarized in Tables S2 and S3.

Table S2: Sensitivity analysis of the ringing measurement loss coefficient $\lambda_{r\ ing}$ (base value: 5×10^{-8}).

λ_{ring}	Relative Factor	PSNR (dB)	SSIM
5×10^{-9}	0.1×	34.46	0.9365
2.5×10^{-8}	0.5×	35.02	0.9422
5×10^{-8}	**1× (chosen)**	**36.40**	**0.9503**
2.5×10^{-7}	5×	35.78	0.9387
5×10^{-7}	10×	35.05	0.9232

Table S3: Sensitivity analysis of the LMG loss coefficient λ_{lmg} (base value: 2×10^{6}).

λ_{lmg}	Relative Factor	PSNR (dB)	SSIM
2×10^{5}	0.1×	35.28	0.9333
1×10^{6}	0.5×	35.84	0.9390
2×10^{6}	**1× (chosen)**	**36.95**	**0.9531**
1×10^{7}	5×	36.01	0.9403
2×10^{7}	10×	35.37	0.9366

In both cases, the peak PSNR and SSIM occur at or very near the chosen coefficients. The PSNR varies by less than 1.2 dB for λ_{ring} and less than 0.7 dB for λ_{lmg} over the full tested range (two orders of magnitude), indicating that the model performance is stable within a reasonable neighborhood around the selected values. These results confirm that the weights determined by the magnitude-balancing strategy are near-optimal and that no extensive grid search is necessary.

S4. Additional Real-World Validation Scenes

To further demonstrate the practical utility of RPCR-Net beyond the two scenes presented in the main manuscript, we conducted additional outdoor experiments at the Siping Road campus of Tongji University under varied illumination and scene conditions. Three representative scenes are shown in Figure S1. For each scene, we present the raw captured image, the restoration result from the baseline learned Wiener-based FOV-KPN, and the result from RPCR-Net, along with an enlarged view of a typical ringing-prone region.

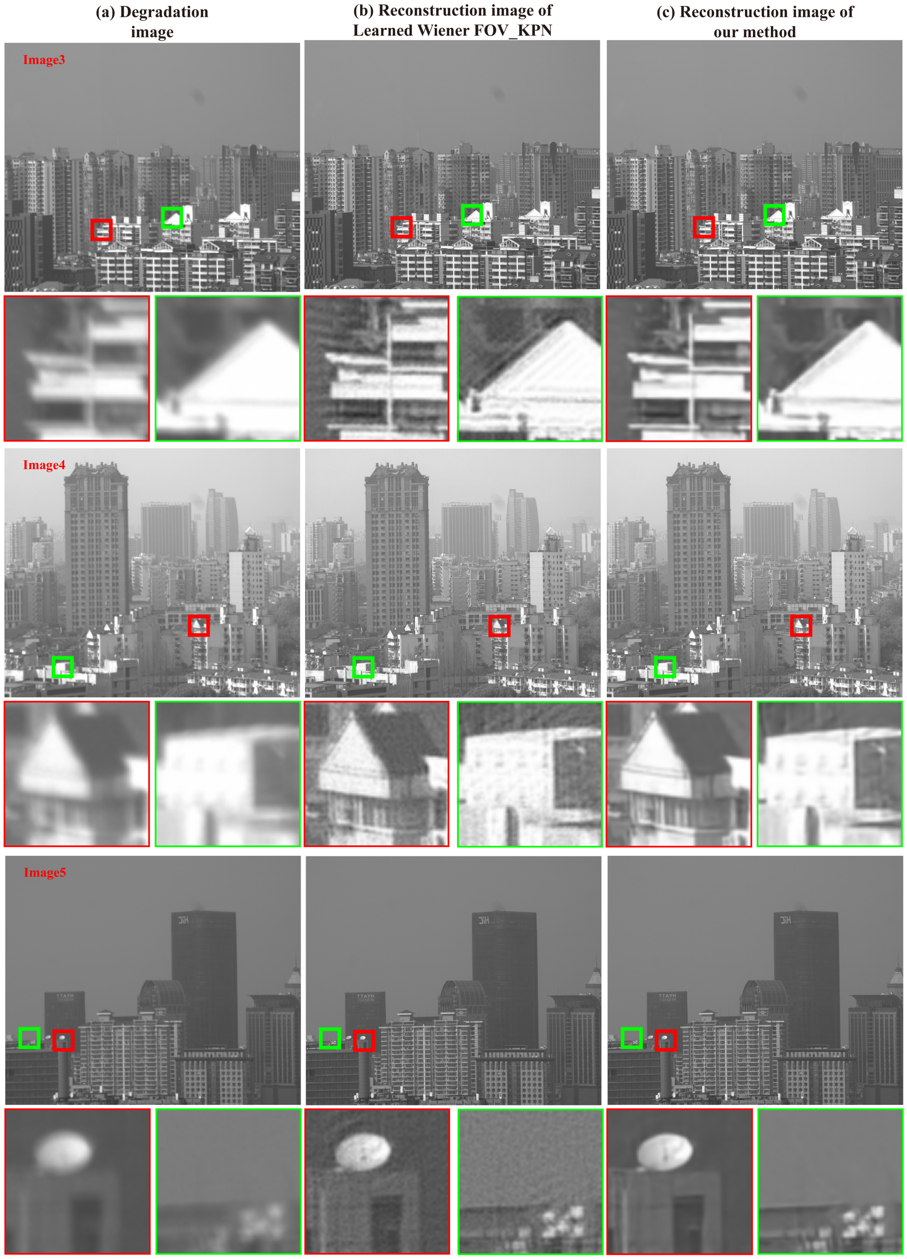

Figure S1: Real-world imaging results on three additional outdoor scenes. (a)–(c) Three distinct scenes captured under different conditions (varying time of day, scene structures, and degrees of overexposure). For each scene, the left panel shows the raw image, the middle panel shows the baseline reconstruction, and the right panel shows the RPCR-Net reconstruction. Enlarged views of regions prone to ringing artifacts are provided below each result. RPCR-Net consistently suppresses ringing artifacts while preserving natural textures and fine details across all scenes.

Together with the two scenes in the main manuscript (Figure 8 and the Supplemental Material of the original submission), the complete evaluation covers **five distinct real-world scenes**. Across all five scenes, RPCR-Net consistently suppresses ringing artifacts and restores clean image details, confirming its effectiveness and robustness under practical imaging conditions.

S5. Pseudocode of the RPCR-Net Training Procedure

For clarity and to facilitate re-implementation, Algorithm S1 summarizes the training step of RPCR-Net. The complete, runnable source code is publicly available at the repository referenced in the Data Availability Statement of the main manuscript.

Algorithm S1 Training Step of RPCR-Net

Require: Overexposed image I_{oe}, ground truth I_{gt}, PSF, noise parameters
Ensure: Updated network parameters θ
  Hyperparameters: $\lambda_{\text{perc}}, \lambda_{\text{ring}}, \lambda_{\text{lmg}}$, learning rate η, L (number of scales for ringing measurement)

$\hat{I} \leftarrow \text{FOV\_KPN}(I_{\text{oe}})$ ▷ Forward reconstruction
$\mathcal{L}_{\text{mse}} \leftarrow \|\hat{I} - I_{\text{gt}}\|_2^2$
$\phi_{\text{pred}} \leftarrow \text{VGG19\_relu2\_2}(\hat{I})$
$\phi_{\text{gt}} \leftarrow \text{VGG19\_relu2\_2}(I_{\text{gt}})$
$\mathcal{L}_{\text{perc}} \leftarrow \|\phi_{\text{pred}} - \phi_{\text{gt}}\|_2^2 \,/\, (C \times H \times W)$
$E \leftarrow 0$
for $l = 0$ **to** $L - 1$ **do**
  if $l > 0$ **then**
    $\hat{I}_l \leftarrow \text{downsample}(\hat{I}, 2^l)$
    $I_{\text{gt},l} \leftarrow \text{downsample}(I_{\text{gt}}, 2^l)$
  else
    $\hat{I}_l \leftarrow \hat{I}$
    $I_{\text{gt},l} \leftarrow I_{\text{gt}}$
  end if
  $D_h \leftarrow |H_h * \hat{I}_l| - |H_h * I_{\text{gt},l}|$
  $D_v \leftarrow |H_v * \hat{I}_l| - |H_v * I_{\text{gt},l}|$
  $\delta_l \leftarrow \text{ReLU}(D_h) + \text{ReLU}(D_v)$
  $E \leftarrow E + \sum_{\text{pixels}} \delta_l$
end for
$\mathcal{L}_{\text{ring}} \leftarrow E$
$G_{\text{pred}} \leftarrow \text{Sobel\_gradient\_magnitude}(\hat{I})$
$G_{\text{gt}} \leftarrow \text{Sobel\_gradient\_magnitude}(I_{\text{gt}})$
$\text{LMG}_{\text{pred}} \leftarrow \text{local\_max\_per\_channel}(G_{\text{pred}}, \text{patch\_size} = 3)$
$\text{LMG}_{\text{gt}} \leftarrow \text{local\_max\_per\_channel}(G_{\text{gt}}, \text{patch\_size} = 3)$
$\mathcal{L}_{\text{lmg}} \leftarrow \|\text{LMG}_{\text{pred}} - \text{LMG}_{\text{gt}}\|_2^2 \,/\, N$
$\mathcal{L}_{\text{total}} \leftarrow \mathcal{L}_{\text{mse}} + \lambda_{\text{perc}} \cdot \mathcal{L}_{\text{perc}} + \lambda_{\text{ring}} \cdot \mathcal{L}_{\text{ring}} + \lambda_{\text{ring}} \cdot \lambda_{\text{lmg}} \cdot \mathcal{L}_{\text{lmg}}$
$\theta \leftarrow \theta - \eta \cdot \nabla_\theta \mathcal{L}_{\text{total}}$

S6. Generalization Validation on an External Dataset

To further substantiate the generalization capability of RPCR-Net beyond the HDREye source images, we conducted an additional evaluation on a completely independent external dataset. We selected 30 images from the **Flickr2K** dataset (Lim et al., CVPRW 2017), which contains diverse high-resolution natural scenes entirely distinct from those in HDREye. Using the identical pipeline described in Section 4.1 of the main manuscript (same optical PSFs, Gaussian–Poisson noise parameters $\sigma_g = 1.1 \times 10^{-3}$ and $\sigma_p = 8.8 \times 10^{-5}$, and scaling factor σ randomly sampled from $[1.1, 1.4]$), we synthesized 30 overexposed test images. The trained

RPCR-Net and the baseline learned Wiener-based FOV-KPN models (originally trained solely on HDREye) were directly applied to these images without any fine-tuning.

Table S4: Quantitative results on the external Flickr2K test set (30 images).

Method	PSNR (dB)	SSIM	LPIPS ($\times 10^{-4}$)	NIQE
learned Wiener-based FOV-KPN	39.21 ± 4.33	0.9713 ± 0.0168	1.150 ± 0.335	3.95 ± 0.50
**RPCR-Net**	**42.37 ± 5.32**	**0.9898 ± 0.0073**	**1.511 ± 0.490**	**3.61 ± 0.68**

As shown in Table S4, RPCR-Net significantly outperforms the baseline FOV-KPN on this completely unseen dataset, improving the PSNR by 3.16 dB and the SSIM by 0.0185.The consistent performance gap between RPCR-Net and the baseline on both the original HDREye test set and the external Flickr2K set confirms that the proposed ringing-perceptive loss function enables genuine generalization rather than overfitting to the training data distribution.

S7. Perceptual Quality and Statistical Analysis

To provide a comprehensive evaluation of reconstruction quality, we report the full set of metrics including LPIPS and NIQE, as well as the standard deviations of PSNR, SSIM, LPIPS, and NIQE over the test set. The complete results for the method comparison and the ablation study are presented in Tables S5 and S6, respectively.

S7.1. Method Comparison with Perceptual Metrics

As shown in Table S5, our RPCR-Net achieves the lowest LPIPS (1.542×10^{-4}) and the lowest NIQE (4.15), demonstrating superior perceptual similarity to the ground truth and better naturalness. Notably, the standard deviation of PSNR for our method (4.00 dB) is smaller than that of UFormer (4.82 dB) and other competitors, indicating more consistent performance across different scenes. The reduced variance in SSIM (0.0188) further confirms the robustness of our approach.

To assess whether the improvements over the baseline (Learned Wiener-based FOV-KPN) are statistically significant, we conducted paired t-tests on all four metrics across the 100 test images. The results confirm a highly significant advantage: PSNR ($t(99) = 6.72, p = 1.17 \times 10^{-9}$), SSIM ($t(99) = 5.38, p = 4.92 \times 10^{-7}$), LPIPS ($t(99) = -6.76$, $p = 9.80 \times 10^{-10}$), and NIQE ($t(99) = 5.89$, $p = 5.32 \times 10^{-8}$). All p-values are far below the 0.001 threshold, demonstrating that the performance gain is not due to random chance.

Table S5: **Full comparison including LPIPS, NIQE, and standard deviations**. Values are reported as mean ± standard deviation over the test set. Best results are in bold.

Method	PSNR (dB)	SSIM	LPIPS ($\times 10^{-4}$)	NIQE
Blurred	29.08 ± 4.44	0.8795 ± 0.0754	8.809 ± 4.100	4.79 ± 1.31
LMG Prior (Chen et al.)	30.06 ± 4.35	0.8965 ± 0.0682	3.325 ± 1.306	4.75 ± 1.39
Ringing Measurement (Mosleh et al.)	29.99 ± 4.37	0.8954 ± 0.0671	3.200 ± 1.298	4.79 ± 1.45
NAFNet	30.67 ± 4.75	0.8987 ± 0.0660	2.709 ± 1.635	4.28 ± 1.27
UFormer	36.95 ± 4.82	0.9501 ± 0.0220	2.361 ± 0.987	4.84 ± 1.44
Restormer	32.17 ± 4.16	0.9500 ± 0.0184	1.851 ± 0.964	3.57 ± 1.54
Learned Wiener-based FOV-KPN	34.76 ± 3.95	0.9459 ± 0.0220	2.701 ± 1.120	4.47 ± 1.37
**RPCR-Net (Ours)**	**37.06 ± 4.00**	**0.9549 ± 0.0188**	**1.542 ± 1.038**	**4.15 ± 1.42**

S7.2. Ablation Study with Perceptual Metrics

The ablation study in Table S6 reveals that the progressive inclusion of the ringing measurement loss and the LMG loss not only improves PSNR and SSIM but also consistently reduces LPIPS and NIQE. The full RPCR-Net achieves an LPIPS of 1.542×10^{-4}, which is substantially lower than the baseline (2.701) and the single-loss variants (2.102 and 1.959). This confirms that the synergistic combination of the two priors yields perceptually more faithful reconstructions. Furthermore, the standard deviation of PSNR for RPCR-Net is 4.00 dB, comparable to the single-loss variants and slightly better than the baseline, indicating that the performance gain does not come at the expense of stability.

Table S6: **Ablation study with LPIPS, NIQE, and standard deviations**. "With Ring" denotes addition of the ringing measurement loss, "With LMG" denotes addition of the LMG prior loss. Values are mean ± standard deviation.

Method	Ring	LMG	PSNR (dB)	SSIM	LPIPS ($\times 10^{-4}$)	NIQE
Blurred	×	×	29.08 ± 4.44	0.8795 ± 0.0754	8.809 ± 4.100	4.79 ± 1.31
Learned Wiener-based FOV-KPN	×	×	34.76 ± 3.95	0.9459 ± 0.0220	2.701 ± 1.120	4.47 ± 1.37
With Ring	✓	×	36.08 ± 3.91	0.9471 ± 0.0235	2.102 ± 0.364	4.18 ± 1.47
With LMG	×	✓	36.35 ± 3.82	0.9482 ± 0.0233	1.959 ± 1.324	4.17 ± 1.30
**RPCR-Net (Ours)**	✓	✓	**37.06 ± 4.00**	**0.9549 ± 0.0188**	**1.542 ± 1.038**	**4.15 ± 1.42**

In summary, the supplementary metrics demonstrate that RPCR-Net not only leads in distortion-based measures (PSNR, SSIM) but also in perceptual quality (LPIPS, NIQE), with statistically significant improvements over the baseline, and exhibits lower or comparable variance, underscoring its effectiveness and robustness for overexposed image reconstruction.

S8. Data Availability

The complete source code of RPCR-Net, the trained model weights, the constructed overexposed image dataset (including the exact training/test split and the random seed), and a README file with detailed instructions for environment setup, training, and evaluation are publicly available at: https://github.com/Ydh-wshxyz/A-Deep-Reconstruction-Framework-with-Ringing-Artifact-Suppression-for-Overexposed-Image.
The original HDREye dataset is available at https://www.epfl.ch/labs/mmspg/downloads/hdr-eye.
